# Military personnel’s satisfaction with antiretroviral services in Namibia: A cross-sectional study

**DOI:** 10.4102/jphia.v16i1.742

**Published:** 2025-02-06

**Authors:** Nestor P.N. Tomas, Nabot P. Kandjimi

**Affiliations:** 1 Department of General Nursing Science, Faculty of Health Science and Veterinary Medicine, University of Namibia, Rundu, Namibia

**Keywords:** antiretroviral therapy, healthcare services, HIV infections, military hospital, Namibia, patient satisfaction

## Abstract

**Background:**

Patient satisfaction is an indicator of healthcare quality and may affect treatment adherence. It is particularly relevant for military personnel living with human immunodeficiency virus (HIV) because of their increased risks to deployment. However, this phenomenon remains understudied.

**Aim:**

To assess satisfaction among military personnel at an antiretroviral clinic in Namibia.

**Setting:**

Military’s antiretroviral clinic in Namibia.

**Methods:**

A descriptive cross-sectional online survey was conducted using a validated third-generation short version of the Patient Satisfaction Questionnaire (PSQ-18). Data were collected between June 2022 and August 2022 from 166 participants through convenience sampling. Bivariable and multivariable logistic regression analyses were performed to identify factors. Adjusted odds ratios (AORs) with a 95% confidence interval (CI) and a *p*-value of 0.05 were used to determine significance.

**Results:**

One hundred and sixty-six participants participated in this study. Significant factors influencing satisfaction included sex, age, marital status and place of residence. Individuals aged 31–44 years were 40 times more likely to be satisfied (AOR: 40.64; 95% CI: 14.16–116.60), while females (AOR: 0.25; 95% CI: 0.16–0.40), divorced individuals (AOR: 0.05; 95% CI: 0.02–0.12) and those residing in urban areas (AOR: 0.33; 95% CI: 0.17–0.62) were less likely to report satisfaction.

**Conclusion:**

The study found a high overall level of patient satisfaction. Based on the results, the study recommends using the Namibia case study to develop and tailor initiatives that improve patient satisfaction with antiretroviral therapy (ART) services among military personnel in other settings.

**Contribution:**

To add to the literature on patient satisfaction with antiretroviral therapy services among military individuals in Namibia.

## Introduction

As of 2020, 37.7 million people were reported to be living with human immunodeficiency virus (HIV) and/or acquired immunodeficiency syndrome (AIDS) globally, including an estimated 10.2m who were not on HIV treatment.^1^ In addition to their health needs, people living with HIV have social and welfare needs that must be managed. Several African nations, including South Africa, have developed policies to mitigate patients’ vulnerability and the impact of HIV and/or AIDS among armed forces.^2^ Central to mitigating the vulnerability of HIV-positive patients, satisfaction with HIV services is essential for promoting the behavioural changes necessary for treatment adherence.^1,3^ Thus, it can serve as a key indicator for evaluating the quality of HIV-related services.^3^ Because of the subjective nature of satisfaction, establishing a universally accepted definition presents challenges. Nonetheless, a common element in these definitions is the assessment of the level of healthcare received, in conjunction with the affective response reflecting the disparity between patients’ expectations and the care they received.^3,4^

Satisfaction is influenced by various factors such as a lack of treatment for opportunistic infections, waiting times, poor management of medication side effects, ineffective communication, and inadequate patient involvement in decision-making.^1^ Somi et al.^5^ linked a connection between satisfaction and personnel competency, the availability of healthcare services and the cost of care. Farooq et al.^6^ considered other factors including socio-demographic characteristics such as health, gender, age and educational status. A low patient satisfaction level can negatively impact the patient’s health quality, resulting in decreased adherence to treatment plans.^1,3^ Military culture, individual belief systems, norm adherence and a lack of mental health services within military environments,^7,8,9^ may lead to elevated levels of psychological distress among military personnel and low life satisfaction.^7^ Consequently, for people living with HIV and/or AIDS, low satisfaction levels may result in reduced engagement with their antiretroviral therapy (ART).^3,7^ Carney et al.^10^ emphasised that mental health conditions such as depressive disorders influence ART adherence among military personnel.

While patient satisfaction is essential to predict adherence,^3,5,7^ there are limited studies that focus on satisfaction with ART services among military individuals. A study by Farooq et al.^6^ noted that patient satisfaction is more likely to be high for the patients receiving ART services at private facilities than those attending at public health facilities.

Despite research suggesting that patient dissatisfaction is the leading cause of treatment defaults, and reduced service utilisation,^1,3^ few studies specifically examined patient satisfaction among military personnel.^6,11^ Furthermore, satisfaction is a subjective phenomenon that may be influenced by variations in contexts, and perception.^6,12,13^ According to Asamrew et al.,^14^ there are two unique dimensions involved in patient assessments of hospital care which include healthcare providers’ interpersonal and technical skills. However, Shaahu et al.^15^ suggested many other dimensions such as patient satisfaction, communication, finance, time spent with a health care provider and access and convenience. However, there is paucity of evidence of validated tools to measure satisfaction in Southern Africa.^16^

Data on the prevalence of HIV among military personnel in Namibia are not publicly accessible. However, literature indicates factors such as deployment increase the risk of HIV among military personnel.^2,17^ Existing evidence indicates that some individuals in Africa prefer traditional treatments to conventional medicine for various diseases, including HIV.^18^ Consequently, patient preferences regarding treatment options may significantly impact their overall satisfaction. Although there is emerging evidence from studies^1,3^ on possible factors for disengagement from HIV care, the measurement of patient satisfaction among military personnel remains rare in Africa.^16^ Many studies investigate general patient satisfaction,^5,16,19,20^ primarily focusing on the general population. However, only a limited number of studies examine satisfaction with HIV services among military personnel,^6,7^ and all of these studies were conducted outside the borders of Africa. Therefore, to ensure that patients’ expectations are met, it is important to measure satisfaction from time to time. Given the identified literature gaps, assessing patient satisfaction will contribute to the improvement of service provision and the enhancement of patient relationships in Namibia. Assessing the satisfaction levels among HIV-positive military personnel can aid in the implementation of comprehensive and sustainable HIV and/or AIDS targeted interventions and policies. This study aimed at assessing the satisfaction levels among military personnel at an antiretroviral (ARV) clinic of a military hospital in Namibia.

## Research methods and design

### Design and setting

This study employed a quantitative descriptive online survey to collect data from people living with HIV and/or AIDS at an ARV clinic of a military hospital in Namibia. This design has been employed to successfully investigate the construct of patient satisfaction in previous studies.^6,11,12^ The hospital is one of the largest military state-funded hospitals in Namibia, and houses about 4000 soldiers in its different units, such as ART, emergency, outpatient, inpatient, and tuberculosis unit. The hospital is staffed by nurses, doctors, pharmacists and social workers who provide free medical services to people living with HIV and/or AIDS. Within the Military Action and Prevention Programme framework, various stakeholders, including the Ministry of Health and Social Services and non-governmental organisations such as the Society for Family Health, are involved in staff training in HIV prevention, care and support.^21^ The hospital was selected for its status as the largest military facility providing ART services to military personnel in Namibia. Patients can visit any health facility of their choice, with various units, including the ARV clinic, available to ensure that all soldiers have access to a comprehensive range of healthcare services.

### Population and sample

The target population comprised all military individuals living with HIV, while the sample pospulation comprised 166 individuals receiving treatment at an ARV clinic of a military hospital in Namibia, recruited through convenience sampling. The sample size was calculated using Solvin’s formula:


$$N/{\left(1+\text{Ne}2\right)}^{22}$$ [Eqn 1]

Where *N* = total population, *n* = sample size, *e* = total confidence limit at 5%. Sample size consisted of 166 patients living with HIV and/or AIDS. This population is adequately representative of your target.^23^ The eligibility criteria were: (1) being a soldier living with HIV and/or AIDS at the selected military hospital; (2) being on ARV treatment and (3) being willing to participate in the study. The study excluded eligible participants who did not turn up during the data-collection period.

### Measures

The study adopted a 36-item Patient Satisfaction Questionnaire (PSQ-18), third-generation short version, an internationally validated tool developed by Farooq et al.^6^ to assess patient satisfaction at a military hospital in Lahore, Pakistan. The tool is freely available to the public and did not require authorisation for modifications to the original version. The inter-item correlation coefficient of the original (PSQ-18) is reported to be between α = 0.64 and 0.77 across the seven domains.^24^ We conducted a reliability test on our adopted tool and obtained a Cronbach’s Alpha score exceeding the acceptable threshold of α = 0.7.^16^

Satisfaction was measured using seven domains. The general satisfaction domain assessed participants’ overall satisfaction through six items (α = 0.96), including questions such as, ‘Are ART clinic staff available during working hours, and is the health centre and its rooms clean?’ The technical quality of services was evaluated using three items (α = 0.97), with inquiries like, ‘Is the service equipment always available and functional?’ Interpersonal manners were measured through five items (α = 0.97), including questions such as, ‘Do health professionals demonstrate compassion and support for patients?’ Communication skills were assessed using four items (α = 0.96), with questions like, ‘Do health workers provide explanations to patients when necessary?’ The finance domain consisted of two items (α = 0.95) aimed at measuring satisfaction with service costs, asking, ‘Have I not paid for the service, and can I afford the medical care I need?’ The time spent with healthcare workers was evaluated using three items (α = 0.92), including questions such as, ‘Am I satisfied with the time spent consulting the nurse, pharmacist, or doctor?’ Lastly, the accessibility and convenience of services were assessed using five items (α = 0.97), for instance, by asking, ‘Is drug availability sufficient during my visit?’

Participants were required to indicate their level of agreement with statements using a 5-point Likert scale (1 = strongly disagree to 5 = strongly agree) in order to assess patient satisfaction. To mitigate passive responses, the answers to negatively worded questions were transposed prior to data analysis.^24^ The scores ranged between 36 and 180, with the maximum indicating the highest degree of satisfaction and vice versa. Responses were further classified according to frequency and percentage, with 80% – 100% (mean score 4–5) being described as satisfied and < 79% (mean score 1–3.9) being classified as dissatisfied.

### Data-collection procedures

After permission was granted by the University of Namibia and the Ministry of Health and Social Services, the researcher approached potential respondents as they came to the ART clinic for follow-up appointments. The participants in the study were required to provide written consent by selecting the ‘agreement button’ in the hyperlink before proceeding to the research questions. To ensure sufficient time for the researcher to engage with participants during their follow-up appointments, data collection commenced in June 2022 and was completed in August 2022.

### Data analysis

Data from the survey questionnaires were analysed using Statistical Package for Social Sciences (SPSS) version 27.0. Descriptive statistics were used to present the data characteristics as percentage, mean and standard deviation (s.d.). To calculate the overall mean satisfaction score, we averaged the scores of each domain. Statistically significant factors with a *p*-value of 0.05 or less from the bivariable and multivariable logistic regression were considered to influence patient satisfaction. We reported adjusted odds ratios (AORs) with a 95% confidence interval (CI) and a *p*-value. The AOR was employed to evaluate the strength of the association between the dependent variable and the independent variables.

### Ethical considerations

Ethical approval for the study was granted by the University of Namibia’s Research Committee (Ref: SoN 99/22) and the Ministry of Health and Social Services (MoHSS) (Ref: 17/1/3/NPK), after which the researcher explained the study’s objectives to the potential respondents. Participation in this study was voluntary, and participants were informed that they could withdraw from the study at any time without any penalties. Privacy was ensured by allowing the respondents to complete the questionnaire in the comfort of their own homes, while confidentiality and anonymity were maintained through password protection and by not linking data to any identifiable personal identities. The electronic data collected were accessible only to the researchers. This study upheld the principles of the revised Declaration of Helsinki.

## Results

### Socio-demographic information

As shown in Table 1, the mean age of the participants was 16.75 years (s.d. = 20.08). Of the respondents, 66% (*n* = 110) were male and 34% (*n* = 56) were female, with ages ranging from 28 years to 57 years. Additionally, 45% (*n* = 75) of the participants were single, 38% (*n* = 63) were married, 9% (*n* = 15) were divorced and 8% (*n* = 13) were widowed. More than half of the respondents (51%, *n* = 84) lived on the military base, 31% (*n* = 52) resided in informal settlements and 18% (*n* = 30) were from the town.

**TABLE 1:** Socio-demographic information.

Variables	Frequency (*n*)	%
**Gender**		
Male	110	66
Female	56	34
**Age (years)**		
Less than 30	8	5
31–44	51	31
45–54	61	37
55 and above	46	28
**Marital status**		
Single	75	45
Married	63	38
Divorced	15	9
Widowed	13	8
**Residence**		
Military base	84	51
Informal settlements	52	31
Town	30	18

Note: Mean age = 16.75 ± 20.08.

### Distribution of patient satisfaction

Table 2 illustrates that the overall mean satisfaction across the seven domains was high at 4.15 (s.d. = 1.00). Satisfaction levels were notably high in the following areas: interpersonal manners (87%, mean [M] = 4.20, s.d. = 1.09), communication (87%, M = 4.17, s.d. = 1.12), finance (87%, M = 4.26, s.d. = 1.15), time spent with healthcare workers (87%, M = 4.17, s.d. = 1.15), access and convenience (87%, M = 4.22, s.d. = 1.09), general patient satisfaction (83%, M = 4.07, s.d. = 1.178) and technical quality (81%, M = 3.99, s.d. = 1.17).

**TABLE 2:** Distribution of patient satisfaction.

Domains of satisfaction	Mean score	s.d.	*n*	%
**Overall mean satisfaction**	4.15	1.00	-	-
**General patient satisfaction**	-	-	-	-
Satisfied	-	-	138	83
Dissatisfied	-	-	28	17
Mean satisfaction	4.07	1.17	-	-
**Technical quality satisfaction**				
Satisfied	-	-	134	81
Dissatisfied	-	-	32	19
Mean satisfaction	3.99	1.17	-	-
**Interpersonal manners**				
Satisfied	-	-	145	87
Dissatisfied	-	-	21	13
Mean satisfaction	4.20	1.09	-	-
**Communication**				
Satisfied	-	-	144	87
Dissatisfied	-	-	22	13
Mean satisfaction	4.17	1.12	-	-
**Finance aspects**				
Agreed	-	-	145	87
Disagreed	-	-	21	13
Mean satisfaction	4.26	1.15	-	-
**Time spent with healthcare workers**				
Satisfied	-	-	144	87
Dissatisfied	-	-	22	13
Mean satisfaction	4.17	1.15	-	-
**Access and convenience**				
Satisfied	-	-	145	87
Dissatisfied	-	-	21	13
Mean satisfaction	4.22	1.09	-	-

s.d., standard deviation.

### Factors associated with patient’s satisfaction

In a logistic regression analysis presented in Table 3, sex, age, marital status and residence were identified as significant variables influencing patient satisfaction with the provision of ARV treatment. Female patients exhibited a 75% decrease in satisfaction (AOR: 0.25; 95% CI: 0.16–0.40) compared to their male counterparts. Patients aged 31–44 years were found to be 40 times more likely to report satisfaction (AOR: 40.64; 95% CI: 14.16–116.60). Furthermore, divorced patients demonstrated a 95% reduction in satisfaction (AOR: 0.05; 95% CI: 0.02–0.12) when compared to those who were married or widowed. Residence also emerged as a critical factor affecting patient satisfaction; patients residing in urban areas reported a 67% lower level of satisfaction (AOR: 0.33; 95% CI: 0.17–0.62) compared to those living in other places.

**TABLE 3:** Regression analysis on the relationship between patient satisfaction and demographic factors (*N* = 166).

Variables	*n*	COR	*p*	95% Cl	AOR	*p*	95% Cl
**Sex**							
Male	110	1.00	-	-	-	-	-
Female	56	3.86	-	2.44–6.08	0.25	0.00	0.16–0.40
**Age (years)**							
< 30	8	1.00	-	-	-	-	-
31–44	51	0.11	0.00	0.00–0.07	40.64	0.00	14.16–116.60
45–54	61	0.76	0.18	0.41–1.18	1.43	0.18	0.84–2.42
˃ 55	46	1.52	0.04	1.02–3.02	0.56	0.04	0.33–0.97
**Marital Status**							
Single	75	1.00	-	-	-	-	-
Married	63	1.35	0.14	0.88–2.27	0.70	0.14	0.43–1.13
Divorced	15	6.16	0.00	7.95–39.11	0.05	0.00	0.02–0.12
Widowed	13	1.17	0.59	0.46–3.80	0.75	0.59	0.26–2.14
**Residence**							
Military base	84	-	-	-	-	-	-
Informal settlements	52	2.25	0.00	1.60–4.25	0.38	0.00	0.23–0.62
Town	30	2.07	0.00	1.59–5.67	0.33	0.00	0.17–0.62

COR, crude odds ratio; AOR, adjusted odds ratio; CI, confidence interval.

## Discussion

Overall, this study indicates that participants were satisfied with ART services. These findings align with a similar study conducted on the general population, which reported an overall satisfaction rate of 84.70% (95% CI: 81.16–88.24).^6^ While the current study reported a higher satisfaction rate than a previous study^25^ conducted in Nigeria and China, which reported satisfaction levels of less than 70.0%, the satisfaction level in this study is lower than that of Mukamba et al.^16^ and Matebu et al.,^19^ which reported satisfaction rates of 74.1% and 89.6%, respectively. The higher satisfaction reported by Ogbo-Okeke et al.^25^ may be attributed to variations in study settings and the effectiveness of the respective countries’ HIV responses and action plans. Lee et al.^26^ suggest that improved delivery of pre-exposure prophylaxis (PrEP) and prevention of mother-to-child transmission (PMTCT) interventions can lead to positive clinical outcomes, such as viral suppression, potentially enhancing patient satisfaction. In Namibia, the rollout of the Military Action Prevention Programme has been noted.^21^ Reasons for higher satisfaction levels in other studies compared to this recent one may be because of the fact that those studies^16,19^ were conducted with civilians rather than military personnel.

The ART clinic staff’s interpersonal skills were desirable, leading to high patient satisfaction in the present study. High ratings for patient satisfaction with staff’s interpersonal manners have been reported in a previous study.^27^ Atsebeha and Chercos^20^ echoed that caring attitudes, good communication and respect shown by healthcare providers towards patients are vital and can help bring about satisfaction among service users. Given that communication is a part of interpersonal skills, the results signify the role communication plays in effectively conveying information in a respectful and friendly manner.^3,19^

With regard to the financial aspects, this study’s results demonstrated a high satisfaction mean score (4.26 ± 1.149) with the affordability of healthcare cost involved. In support of the present study’s findings on financial aspects, a study conducted in Nigeria has found high satisfaction related to affordability.^15^ Similarly, in Farooq et al.’s^6^ study, the respondents report satisfaction (mean score 4.20 ± 1.086) with the financial aspect of their healthcare. Satisfaction was associated with the availability of affordable or free public health services.^4,6^ In terms of time spent, our study found a higher mean patient satisfaction with the time spent with healthcare workers in the ART clinic setting, compared to the recent mean satisfaction scores of 3.22 and 3.28 reported in the literature.^4,6^

A logistic regression analysis identifies several factors that significantly impact patient satisfaction with ART services. Age group emerged as a significant determinant of patient satisfaction in this study, with patients aged between 31 and 40 years reporting higher levels of satisfaction. This finding contrasts with a study conducted on patient satisfaction with HIV services in the general population, which indicated dissatisfaction among both males and females.^5^ The discrepancies in results may be attributed to variations in the study settings.

Moreover, gender exhibited a statistically significant influence on satisfaction in this study. The analysis revealed that female patients were 75% less satisfied (AOR: 0.25; 95% CI: 0.16–0.40) compared to their male counterparts. These findings are consistent with a study on satisfaction with service delivery among HIV treatment clients, which indicated that females were less likely to be satisfied (AOR 0.52 for 2000–4000 vs. < 2000; 95% CI: 0.30–0.90; AOR 0.50 for > 4000 vs. < 2000; 95% CI: 0.27–0.93).^28^ Furthermore, a study on patient satisfaction in a military hospital in Pakistan found that male respondents were significantly more satisfied than their female counterparts (*p* = 0.05).^6^ In contrast, research conducted in Nigeria by Akunne et al.^29^ revealed that a higher proportion of females (69%) compared to males (31%) reported greater satisfaction with HIV and AIDS care services. Additionally, Mukamba et al.^16^ identified differences in satisfaction levels between males and females; however, no statistically significant difference was observed (*p* = 0.54). The mismatch of males and females’ satisfaction could be attributed to the disparities related to differences in expectations regarding services.^28,30^ Similarly, the differences in satisfaction between males and females could be attributed to the differences in socioeconomic-demographic characteristics, cultural beliefs, increased sexual behaviours and high alcohol consumption among males than females.^2,18,31^

In alignment with the study conducted by Dzamboe et al.,^12^ our findings indicate that participants residing in towns exhibited significantly lower satisfaction levels compared to those in other areas (AOR: 0.33; 95% CI: 0.17–0.62; *p* = 0.00). These results are surprising, as patients in rural areas often exhibit lower literacy levels and health knowledge, which subsequently influences their perception of quality healthcare.^16^ A recent study conducted by Mokhele et al.^28^ found that the odds ratio of patient satisfaction decreases by 33% for individuals in urban healthcare facilities (AOR 0.67; 95% CI 0.46–0.99). However, our results are different from that of Ayele et al.,^32^ which stated that patients living in rural areas reported 3.0 times greater satisfaction (AOR = 3.0; 95% CI: 1.8–5.2) than their urban counterparts. This apparent contradiction in results may be attributed to the speculation that patients in urban areas generally have higher expectations, leading them to anticipate lower service quality.

Clearly, the study results demonstrate a high level of patient satisfaction at a military hospital. Considering that satisfaction is a crucial indicator for evaluating the effectiveness of healthcare service delivery, the findings of the current study are essential for predicting positive service outcomes. This study has added to the literature on patient satisfaction with ART services among military individuals in Namibia. Given political leadership and effective implementation of public health priorities, access to quality and free service is guaranteed in Namibia, making the country to become the first country in the world and in Africa to reach a milestone towards eliminating the HIV burden that mainly includes mother-to-child transmission of both HIV and viral hepatitis B.^33^ This progressive achievement highlights Namibia’s dedication to improving health outcomes and demonstrates the potential for other nations to follow in their footsteps. The study recommends utilising the Namibia case study to develop and adapt initiatives designed to enhance patient satisfaction with ART services among military personnel.

### Limitations

The major limitations of this study were the use of self-reporting data, allowing social desirability bias where participants provided responses that they believe are more socially acceptable rather than expressing their true feelings or experiences. Additionally, the recruitment strategy was limited to only those who adhered to follow-ups and excluded individuals who were lost to follow-up, limiting the generalisability of the findings to the broader population. The use of online survey in this study may not accurately represent participants who lacked access to smartphones. Future studies are needed to investigate the correlation between satisfaction and HIV outcomes, including viral load and medication adherence.

## Conclusion

The study reveals a higher overall level of satisfaction with ART service provision at a military hospital in Namibia. Notably, age was identified as a significant factor, with young adults aged 31–40 years demonstrating greater satisfaction with ART services. Furthermore, female patients reported lower satisfaction levels compared to their male counterparts. High patient satisfaction may be related to political leadership and effective implementation of public health priorities, access to quality and free service is guaranteed in Namibia. Future studies should investigate the correlation between satisfaction and HIV outcomes, including viral load and medication adherence.
